# Sexual quality of life and sexual self-efficacy among women during reproductive-menopausal transition stages and postmenopause: a comparative study

**DOI:** 10.1186/s40695-021-00067-2

**Published:** 2021-09-17

**Authors:** Hedyeh Riazi, Fatemeh Madankan, Seyed Ali Azin, Maliheh Nasiri, Ali Montazeri

**Affiliations:** 1 Department of Midwifery and Reproductive Health, School of Nursing and Midwifery, Shahid Beheshti University of Medical Sciences, Tehran, Iran; 2 Reproductive Biotechnology Research Center, Avicenna Research Institute, ACECR, Tehran, Iran; 3 Department of Basic Sciences, School of Nursing and Midwifery, Shahid Beheshti University of Medical Sciences, Tehran, Iran; 4 Population Health Research Group, Health Metrics Research Center, Iranian Institute for Health Sciences Research, ACECR, Tehran, Iran; 5 Faculty of Humanity Sciences, University of Science and Culture, Tehran, Iran

**Keywords:** Sexual behavior, Self-efficacy, Quality of life, Menopause

## Abstract

Sexual self-efficacy is essential for appropriate and desirable sexual function and sexual quality of life. This study aimed to compare sexual quality of life and sexual self-efficacy among women during reproductive-menopausal transition stages and postmenopause. This was a cross-sectional study of a sample of Iranian women. The sexual quality of life-female (SQOL-F) scale was used to measure sexual quality of life (SQOL) and sexual self-efficacy (SSE) was measured using the sexual self-efficacy questionnaire (SSEQ). Data were compared between the study groups using multiple linear regression. In all 340 women (170 in reproductive-menopausal transition stages and 170 postmenopause) were studied. The mean ages of reproductive-menopausal transition stages and postmenopausal women was 30.8 ± 6.55 and 56.3 ± 3.54 respectively (*P* < 0.001). Sexual self-efficacy and sexual quality of life were found to be significantly higher in reproductive-menopausal transition stages compared with postmenopause women (*P* < 0.001 and *P* = 0.017 respectively). Sexual and relationship satisfaction and sexual repression subscales differed significantly between the two groups (*P* = 0.001 and *P* < 0.001 respectively). Higher sexual self-efficacy contributed to higher sexual quality of life (*P* < 0.0001). Reproductive-menopausal transition stages women appear to enjoy higher levels of sexual self-efficacy and sexual quality of life. Given the importance of sexual quality of life, it is recommended to pay greater attention to sexual self-efficacy among postmenopausal women in order to improve sexual quality of life in this population.

## Background

Sexual relationships, a fundamental dimension of human life, play a key role in the life of most individuals regardless of marital status [1]. These relationships are easily reinforced or inhibited by emotions associated with self-efficacy [2]. Sexual self-efficacy refers to one’s self-confidence for managing sexual relationships and properly adapting to the sex partner [3]. Improving sexual self-efficacy can prevent sexual problems and promote sexual quality of life in women [4]. In fact, sexual self-efficacy is essential for having appropriate and desirable sexual function at different ages [5], which in turn might improve sexual quality of life [6–8].

Sexual quality of life refers to one’s evaluation of the positive and negative aspects of sexual relationships and response to this evaluation [9]. It also refers to the perception of sexual functions [10], predicts the consequences of sexual problems [11, 12], and mental and physical health [13]. Having a desirable level of sexual quality of life is fundamental in sexual and reproductive health, and is associated with improvements in general quality of life [14].

The prevalence of sexual problems is relatively high in Iranian women, ranging from 26 to 51% [13]. Moreover, numerous studies suggest the negative effects of aging and menopause on sexual quality of life in women [15]. Sexual dysfunction is 3.2 times more common in postmenopausal women compared to women in the reproductive-menopausal transition stages [16]. Given a life expectancy of 76 years in Iranian women, they spend more than 20 years of their life after menopause [17]. It is therefore crucial to improve sexual quality of life during this long period. Despite this, the majority of studies conducted on sexual health have focused on sexual function and general quality of life in women [18, 19]. Indeed, one area of missing information is whether women have the same ability and desire for sexual functioning during reproductive-menopausal transition and postmenopausal stages. Thus the present study was conducted to focus on sexual function differences in Iranian women based on their menopausal status as in reproductive or menopausal transition stages vs. postmenopause. To our knowledge, no study has so far been conducted to compare these two groups in terms of sexual self-efficacy or sexual quality of life in the Iranian population.

## Methods

### Study design and participants

The present cross-sectional study recruited a sample of women in reproductive-menopausal transition and postmenopausal. The sample size was estimated using a previous study on sexual self-efficacy and marital satisfaction [20]. Convenience sampling was used to select the women presenting to health centers in Abyek, Iran in 2017. The study included all four health centers in Abyek and based on required sample size, 85 participants were selected from each center. One of us (FM) was present in all centers. She invited women who were attending for routine care to the centers. After permission and consent she interviewed women and collected the data. The general inclusion criteria consisted of being married, healthy, no family disputes in the last month, not experiencing stressful events within the last six months, no drug and alcohol addiction and not taking drugs that affect libido. The particular inclusion criteria comprised the absence of pregnancy and breastfeeding in reproductive-menopausal transition stages women and having elapsed 12 months from their menstruation cessation, menopause being physiologic and not currently undergoing hormone therapy in postmenopausal women. The only exclusion criterion was not responding to all measures.

### Measures

The data collection tools consisted of demographic information, sexuality quality of life-female (SQOL-F) and sexual self-efficacy (SSEQ) questionnaires.

- 1. Sexual Quality of Life-Female (SQOL-F) Questionnaire

In order to evaluate quality of sexual life, the Sexual Quality of Life-Female (SQOL-F) questionnaire designed by Symonds was used [21]. The psychometric properties of the Iranian version of SQOL-F are well documented [10, 12]. It consists of 18 items and each item is rated on a six-point response category (completely agree to completely disagree). The scores on this scale range from 18 to 108 and a higher score indicates better sexual quality of life [21]. It is a self-report instrument and includes four subscales. Examples of items are provided in parentheses: Psychosexual Feelings (measuring anger, worry of partner’s hurt or rejection), Sexual and Relationship Satisfaction (enjoy, good feeling about oneself), Self-worthlessness (feeling like less of a woman, feeling of guilt), and Sexual Repression (loss of pleasure, avoiding) [21].

- 2. Sexual Self-Efficacy Questionnaire (SSEQ)

The 10-item Sexual Self-Efficacy Questionnaire was developed by Vaziri [20] based on the Schwarzer’s general self-efficacy questionnaire [22]. The scores on this questionnaire range from 0 to 30. The reliability of this questionnaire was reported to be 0.86 using Cronbach’s alpha, 0.81 using the split-half method of Spearman-Brown, and 0.81 using Gottman’s method and 0.80 using test–retest analysis [23]. The questionnaire measures items about one’s self confidence in doing sexual activities, sexual competence, and sexual pleasure.

### Statistical analysis

Statistical tests such as chi-squared test and independent t-test were used to compare data between the two study groups. The association between sexual quality of life and independent variables was assessed using linear regression analyses using separate models to assess age versus menopausal status. As such, sexual quality of life was the dependent variable and age, education occupation, income, number of children, and sexual self-efficacy were considered as independent variables. The output then was reported as unstandardized and standardized regression coefficients (β), and p value. The p value of < 0.05 was set as the level of statistical significance.

## Results

In all 340 (170 reproductive-menopausal transition and 170 postmenopause stages) women participated in the study. The mean age was 30.85 ± 6.55 years for reproductive-menopausal transition stage and 56.33 ± 3.54 years for postmenopausal women (*p* < 0.001). The majority of postmenopausal women (48.2%) were illiterate or had primary education while most reproductive-menopausal transition stages women (83%) had higher education. Most participants were housewives. Table 1 presents the characteristics of participants.

**Table 1:** Characteristics of the study samples

	**All (n = 340)**	**Reproductive-menopausal transition stage (n = 170)**	**Postmenopause (n = 170)**	*p* value*
	**No (%)**	**No (%)**	**No (%)**	
**Age**	43.59 ± 13.80	30.85 ± 6.55	56.33 ± 3.54	< 0.001
**Education**				< 0.001
Illiterate/primary	89 (26.2)	7 (4.1)	82 (48.2)	
Secondary	171 (50.3)	94 (55.3)	77 (45.3)	
Higher	80 (23.5)	69 (40.6)	11 (6.5)	
**Occupation**				< 0.001
Housewife	202 (59.4)	79 (46.5)	123 (72.4)	
Employed	138 (40.6)	91 (53.5)	47 (27.6)	
**Income**				< 0.001
Poor	73 (21.5)	14 (8.2)	59 (34.7)	
Intermediate/Good	267 (78.5)	156 (91.8)	111 (65.3)	
**Number of pregnancies**				< 0.001
0–2	166 (48.8)	139 (81.8)	27 (15.9)	
3–4	104 (30.6)	30 (17.7)	74 (43.5)	
5–8	70 (20.6)	1 (0.5)	69 (40.6)	
**Number of children**				< 0.001
0–2	195 (57.3)	160 (94.1)	35 (20.6)	
3–4	90 (26.5)	10 (5.9)	80 (47.1)	
5–8	55 (16.2)	0 (0)	55 (32.3)	

^*^ Derived either from t-test or chi-square

In univariate analysis there were significant differences between reproductive-menopausal transition and postmenopausal women in sexual self-efficacy and sexual quality of life scores. Sexual self-efficacy was greater among the reproductive-menopausal transition stage women than among the postmenopausal women (*p* < 0.001). Comparing the subscales of sexual quality of life between the two groups revealed statistically significant differences for sexual and relationship satisfaction and sexual repression. Sexual and relationship satisfaction was significantly greater among women in the reproductive-menopausal transition stages than the postmenopausal women (*p* = 0.001) (See Table 2). Sexual repression was significantly greater for the postmenopausal women (*p* < 0.001).

**Table 2:** Sexual self-efficacy and sexual quality of life in postmenopause and reproductive-menopausal transition stage women^a^

	**All (n = 340)**	**Reproductive-menopausal transition stage (n = 170)**	**Postmenopause** **(n = 170)**	*p* value**
	**Mean (SD)**	**Mean (SD)**	**Mean (SD)**	
**Sexual self-efficacy**	16.62 (7.81)	20.8 (6.29)	12.4 (6.83)	< 0.001
**Sexual Quality of Life**				
Psychosexual feelings	36.15 (6.45)	36.37 (6.99)	35.93 (5.88)	0.53
Sexual and relationship satisfaction	24.45 (4.88)	25.21 (4.81)	23.69 (3.77)	0.001
Self-worthlessness	16.22 (2.3)	16.27 (2.56)	16.18 (2.10)	0.71
Sexual repression	15.11 (3.46)	16.04 (3.24)	14.19 (3.43)	< 0.001
Total score	91.93 (15.1)	93.88 (6.36)	89.98 (13.36)	0.017

^a^ Higher scores for the scale and the subscales indicate better conditions. For negative concepts that are sexual self-worthlessness and sexual repression higher scores mean less worthlessness and repression

^**^ Derived from t-test

Since reproductive-menopausal transition stage and postmenopausal women differed significantly in demographic, obstetrics and sexual characteristics, the association between sexual quality of life and independent variables including age, education, occupation, income, number of children, and sexual self-efficacy was examined using linear regression analyses. Due to collinearity between age and group, different models were run. The results are presented in Table 3. As shown it was found that in all instances sexual-self-efficacy significantly was associated with sexual quality of life. For instance, an increase of 1 score in sexual self-efficacy was associated with an average increase of 0.75 to 0.76 in sexual quality of life score (model 3a and model 3b, respectively). In addition, the findings revealed that being in the postmenopausal stage decreased the sexual quality of life score by 0.30 (model 3a) while a 1-year increase in age was associated with a decrease of 0.25 in sexual quality of life score.

**Table 3:** Association between sexual quality of life and independent variables obtained from linear regression analyses^a^

**Models**	**Unstandardized coefficients**		**Standardized coefficients**	**t**	***p* value**
	**β**	**Std. Error**	**β**		
***Model 1a**** (adjusted R*^*2*^ = *0.431)*					
Sexual self-efficacy	1.481	0.094	0.769	15,774	< 0.0001
Menopausal vs. non-menopasual (Group)	-8.609	1.464	-0.286	-5.879	< 0.0001
***Model 1b**** (adjusted R*^*2*^ = *0.413)*					
Sexual self-efficacy	1.483	0.102	0.770	14.542	< 0.0001
Age	-0.276	0.058	-0.253	4.782	< 0.0001
***Model 2**** (adjusted R*^*2*^ = *0.107)*					
Education(primary vs. higher)	-2.855	3.363	-0.084	-0.848	0.397
Education (secondary vs. higher)	-0.743	2.289	-0.025	-0.325	0.746
Occupation (housewife vs. employed)	-3.266	1.979	-0.107	-1.650	0.100
Income (poor vs. intermediate/good)	-3.946	2.223	-0.108	-1.775	0.077
Number of children	-1.271	0.598	-0.155	-2.124	0.034
***Model 3a**** (adjusted R*^*2*^ = *0.430)*					
Sexual self-efficacy	1.457	0.105	0.756	13.894	< 0.0001
Menopasual vs. non-menopausal (Group)	-9.193	1.864	-0.306	-4.931	< 0.0001
Number of children	-0.286	0.564	-0.035	-0.507	0.612
***Model 3b**** (adjusted R*^*2*^ = *0.411)*					
Sexual self-efficacy	1.482	0.108	0.769	13.676	< 0.0001
Age	-0.278	0.078	-0.255	-3.565	< 0.0001
Number of children	-0.023	0.608	-0.003	-0.039	0.969

^a^ Dependent variable: sexual quality of life (SQOL)

## Discussion

The present study revealed that postmenopausal women had lower sexual self-efficacy compared to reproductive-menopausal transition stage women. The hope for enjoying sexual life for a longer time appears to be more persistent in reproductive-menopausal transition stages women, which ultimately provides more frequent opportunities for sexual interactions and thereby improves their sexual self-efficacy. One might argue that women differed not only by reproductive-menopausal transition and postmenopausal stages, but also by age and also by generation. Generational differences are related to women's birth cohort. Women’s lives are quite different from generation to generation. As shown in the results, there were dramatic differences in the number of pregnancies and number of children between the reproductive-menopausal transition and postmenopausal groups as well as differences in women’s occupations, education, and income.

It seems that physical and psychological changes occurring after menopause affect women’s perception of their efficacy, capacity and capability in sexual circumstances [16]. Given the close relationship among physical conditions, self-efficacy and emotional disorder, women experience declining perceptions about their own abilities in this period and therefore present lower levels of sexual self-efficacy [24]. In addition, many women are unaware of how to deal with menopause problems and they are unable to cope with stressful situations, which can negatively affect sexual and marital relationships and cause negative feelings in women [25]. Teaching marital and sexual skills to women may be an effective step towards solving women’s sexual problems, since improving sexual self-efficacy can solve fundamental sexual problems in women [26]. Ramezani et al., reported low scores for sexual self-efficacy in Iranian women, which can be explained by the wide range of the women’s age they studied (from 20 to 60) [27]. Besides, they did not compare postmenopause and reproductive-menopausal transition stages women; however, other studies enrolled women of childbearing age, reported high mean scores for sexual self-efficacy, which is consistent with the mean score obtained in the present study in the reproductive-menopausal transition stages women [28, 29].

Based on the findings, reproductive-menopausal transition stages women had significantly higher mean scores of sexual quality of life compared to postmenopausal women, which is consistent with some other studies [14]. Although we did not collect data on specific postmenopause symptoms, evidence suggest that many women face various stressful experiences during postmenopause, including children leaving the home, the need for taking care of old parents, retirement, developing diseases and various physical problems and also a higher prevalence of sexual disorders accompanying aging [24], all of which could reduce their sexual quality of life. Moreover, given the social and cultural background of the Iranian community, postmenopausal women are less inclined to maintain sexual relationships, are less focused on sexuality, and perceive their sexual life as finished, the roots of which can be sought in the family, culture and society [12]. Finding solutions to improve sexual quality of life appears crucial, since it is a major predictor of joy and satisfaction in life [9]. Enjoyable sexual life is an important element in human well-being particularly in the postmenopause period [30].

No statistically significant differences were observed between the two groups in terms of subscales, including psychosexual feelings and self-worthlessness. Psychosexual feelings appear to be associated with feelings such as anxiety, depression, failure and fear of upsetting the sexual partner. Self-worthlessness also appears to be associated with low self-confidence and self-doubt [24]. These are general issues in people’s lives and are not associated with a specific period of one’s life [25].

Statistically significant differences were observed between the two groups in sexual and relationship satisfaction and sexual repression as derived from univariate analysis (t-test). The reproductive-menopausal transition stages women presented higher sexual and relationship satisfaction than the postmenopausal women. Reproductive-menopausal transition stage women may establish more desirable sexual relationships due to paying more attention to and deal more with sexual matters and giving higher priorities to sexual relationships in marital life. Menopause can reduce sexual and relationship satisfaction in women by reducing the level of sexual steroid hormones, and related factors such as hot flashes, vaginal dryness and reduced frequency of sexual intercourse [31]. Thus adopting preventive measures to reduce the number of complications and improve sexual satisfaction is important. Reproductive-menopausal transition stages women were found to have lower sexual repression compared to postmenopausal women, possibly due to cultural effects in the postmenopausal group. Sexual repression is associated with avoiding sexual intercourse and can be affected by cultural factors and social norms. In one study of reproductive-menopausal transition stage women, higher levels of sexual desire and needs was associated with sexual repression and increased frequency of sexual self-blame, internal conflicts, frustration and incompatibility [12].

Higher sexual self-efficacy contributed to higher sexual quality of life. Sexual self-efficacy is vital for having desirable sexual activity [32] and contributed to higher sexual quality of life. Higher level of sexual self-efficacy is associated with the ability to solve sexual problems and improvements in the quality of sexual relationship [7]. Other studies also suggest that sexual self-efficacy is a major predictor of sexual relationships and that negative thoughts about sexual behaviors can cause poor sexual performance and even avoidance of sexual activities, which in turn reduce sexual quality of life [33].

## Limitations

This study had some limitations. Samples were selected from public health centers in Abyek, a city in Qazvin province, so women who did not seek any health care were not included. Also the study sample was not representative of the Iranian population in terms of the socio-economic situation and the geographic variations. Finally, one should note that perhaps the data on sexual issues should be looked at from the perspective of youngest to oldest rather than looking at reproductive-menopausal transition stages and postmenopause groups. That is, to calculate how sexual issues change over the life span of the woman as she ages. We were unable to do so since this would require a larger number in each age group and a different analysis plan.

## Conclusion

The present study demonstrated that sexual self-efficacy and sexual quality of life were higher in reproductive-menopausal transition stages women. Reproductive-menopausal transition stage women appear to enjoy higher levels of sexual self-efficacy and sexual quality of life, due to paying more attention to sexual matters and giving higher priorities to sexual relationships in marital life compared to postmenopausal women. According to the results of the present study better sexual relationship and higher sexual self-efficacy could result in higher sexual quality of life. Given the key role of sexual relationships in marital life, proposing solutions such as training programs should be considered to improve sexual self-efficacy and sexual quality of life and therefore sexual health in women, especially in postmenopause.

## Data Availability

The data sets used and/or analyzed during the current study are available from the corresponding author on reasonable request.

## Abbreviations

SSE: Sexual self-efficacy
SSEQ: Sexual self-efficacy questionnaire
SQOL-F: Sexual quality of life-female
SQOL: Sexual quality of life
